# A birth cohort study of viral infections in Vietnamese infants and children: study design, methods and characteristics of the cohort

**DOI:** 10.1186/1471-2458-13-937

**Published:** 2013-10-08

**Authors:** Katherine L Anders, Nguyet Minh Nguyen, Nguyen Thi Van Thuy, Nguyen Trong Hieu, Hoa L Nguyen, Nguyen Thi Hong Tham, Phan Thi Thanh Ha, Le Bich Lien, Nguyen Van Vinh Chau, Cameron P Simmons

**Affiliations:** 1 Oxford University Clinical Research Unit, Hospital for Tropical Diseases, Ho Chi Minh City, Vietnam; 2 Nuffield Department of Clinical Medicine, Centre for Tropical Medicine, University of Oxford, Oxford, UK; 3 Department of Epidemiology and Preventive Medicine, Monash University, Melbourne, Australia; 4 Hospital for Tropical Diseases, Ho Chi Minh City, Vietnam; 5 Hung Vuong Hospital, Ho Chi Minh City, Vietnam; 6 Dong Thap Hospital, Cao Lanh, Vietnam; 7 District 8 Hospital, Ho Chi Minh City, Vietnam; 8 Department of Dengue Haemorrhagic Fever, Children’s Hospital No. 1, Ho Chi Minh City, Vietnam

**Keywords:** Cohort studies, Epidemiology, Infections diseases, Viral infectious, Infants

## Abstract

**Background:**

In Ho Chi Minh City, Vietnam, more than one-third of admissions to the two paediatric hospitals are attributable to four infectious syndromes: dengue, diarrhoeal disease, acute respiratory infection, and hand, foot and mouth disease. We have established a large prospective birth cohort study to investigate individual, environmental, virological, and immunological determinants of infection and disease in infants. Specific research questions are focused on the role of maternal antibody in protection against infection in infancy, and the adaptive immune response to vaccination and natural infection. This paper presents the cohort design, methods, and baseline characteristics of the participants enrolled in the first two years.

**Methods/design:**

Women are enrolled prior to delivery at one hospital in each of two catchment areas: an urban district in central HCMC, and a mixed urban/rural district in the Mekong Delta 150 km southwest of HCMC. Infants are enrolled within 3 days of birth, and maternal and cord blood samples are collected. Routine blood samples and data on growth, health status and vaccinations are collected from infants at scheduled visits at 4, 9 and 12 months. Clinical data and specimens are collected from infants presenting at a study clinic, or admitted to hospital, with any of the the four infectious syndromes of interest.

**Discussion:**

In four years since since the study began in July 2009, >6400 infants have been enrolled, and enrolment is ongoing. Attrition is low: 84% of participants have completed the full 12-month follow-up period. Baseline characteristics of the first 4300 enrollees are presented here. We have demonstrated the feasibility of establishing a large prospective study of infectious diseases in infancy in a resource-limited setting, with minimal loss to follow-up. Our linked socio-demographic, clinical and laboratory data will help elucidate the viral aetiology and epidemiology of common infectious diseases of infancy, and can inform the implemention of existing and future vaccines. This study furthermore provides a platform to which additional endpoints could be added in the future.

## Background

A number of large birth cohort studies have been established in south east Asia that focus on environmental exposures, child health and development, asthma, allergy and obesity in children, [1-3] however there are few published longitudinal studies of infection and immunity in infants and young children in this region [4-6]. Studies of infectious disease aetiology and epidemiology are often cross-sectional and hospital-based in design, which presents a number of limitations including a focus on more severe illness, a lack of information on pre-infection risk factors, an inability to estimate incidence due to the lack of an appropriate denominator, and an inability to identify asymptomatic infections which may be an important contributor to transmission at a population level. Although a wealth of literature from prospective community-based studies does exist for a large range of infectious diseases which addresses questions of case burden, aetiology, epidemiological risk factors, pathogenesis and immune response, many studies focus only on one pathogen or syndrome and do not therefore give a broad picture of the longitudinal infectious disease experience of participants. Others are limited by small sample size, lack of routine blood sampling for serological detection of asymptomatic infections, or lack of year-round case detection.

In southern Vietnam, more than one-third of admissions to the two referral paediatric hospitals in HCMC are attributable to 4 infectious diseases and syndromes: dengue, diarrhoeal disease, acute respiratory infection (ARI), and hand, foot and mouth disease (HFMD), with an average of 147,000 patients admitted with these syndromes annually to these two hospitals alone (personal communication: Corinne Thompson). The median age of dengue cases in southern Vietnam is 9 years, however hospital-based studies have demonstrated a second peak of hospitalisations for severe dengue among infants aged 4–10 months [7] and that young children <5 years are at higher risk of mortality from dengue than older children [8]. Children under 2 years account for the majority of hospital admissions with diarrhoeal disease [9,10] and ARI, [11] and the median age of children admitted to hospital in HCMC with HFMD was 20 months during a recent epidemic, [12] in which enterovirus 71 emerged as a major pathogen associated with severe disease.

We have established a prospective birth cohort spanning an urban and a semi-rural population in southern Vietnam, that serves as a platform for longitudinal epidemiological, clinical and immunological studies of these four major infectious syndromes in Vietnamese infants and children. The primary objective around which this study was designed was to determine the role of passively acquired maternal antibody in protection against and pathogenesis of dengue virus infection during the first year of life. The secondary objectives were to determine the incidence of acute respiratory infection and gastrointestinal infections in the first year of life, the viral aetiology of those infections, and the risk factors associated with viral infections during infancy.

## Methods/design

### Study population and location

**Figure 1:**
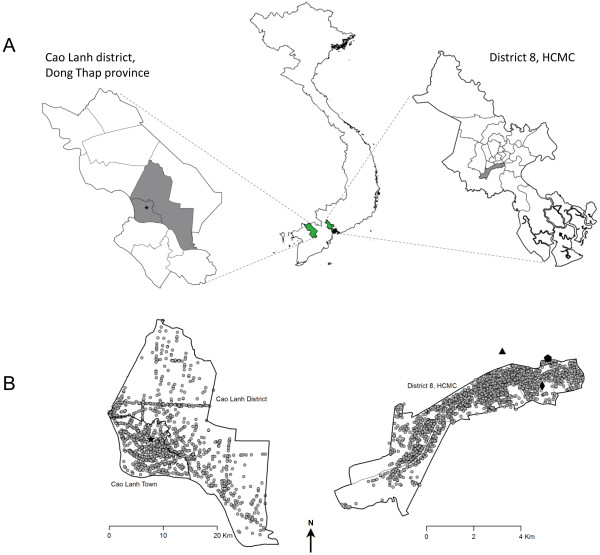
**Study area. A**. The infant cohort is drawn from two catchment populations in southern Vietnam, shaded grey: 1) District 8 in Ho Chi Minh City (HCMC), and 2) Cao Lanh District (including Cao Lanh Town) in Dong Thap Province, showing the location of the collaborating site Dong Thap Hospital (star). **B**. Spatial distribution of the residence of cohort participants in Cao Lanh Town and District, Dong Thap (left) and District 8, HCMC (right). Lines show administrative wards (n = 333 in DT and n = 16 in HCMC) and symbols show location of collaborating hospitals: Dong Thap Hospital (star), Hospital for Tropical Diseases (pentagon), Hung Vuong Hospital (triangle) and District 8 Hospital (diamond).

**Figure 2:**
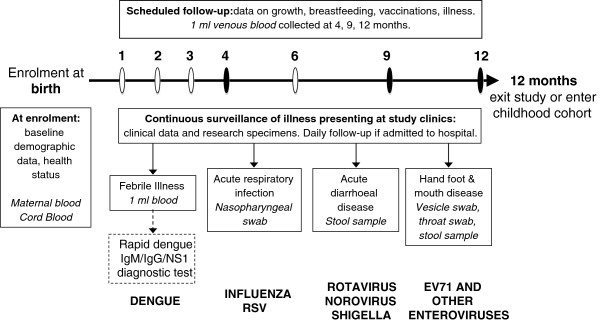
**Study design.** Infants are enrolled at birth and followed until one year of age. Baseline data, cord blood and a maternal blood sample are collected at birth. Data on health and development are collected at scheduled visits at 2, 4, 6, 9 and 12 months of age (in HCMC there are two additional appointments at 1 and 3 months). A one ml blood sample is also collected at the visits at 4, 9 and 12 months. Families are asked to bring the infant to a study clinic with any illness. Clinical data are recorded on examination, and specimens are collected according to the symptoms and presumptive diagnosis. Detailed clinical data are collected daily for any infant admitted to hospital with an infectious disease. The four syndromes on which research studies are focused are febrile illness, acute respiratory infection, acute diarrhoeal disease, and hand foot and mouth disease, and the pathogens for which diagnostics are currently being performed are indicated.

The infant cohort is drawn from two catchment populations (Figure 1). The first is District 8 in Ho Chi Minh City (HCMC), a highly urban, inner-city district with a population in 2009 of 408,772 and a population density of 20,545 per km^2^. This district was chosen for its close proximity to the Hospital for Tropical Diseases (HTD) and because it is over-represented among admissions for dengue, respiratory and gastrointestinal illness at HTD as well as at the main paediatric hospital in HCMC, Children’s Hospital number 1 (CH1). The second catchment is Cao Lanh Township and Cao Lanh District in Dong Thap Province, 150 km south-west of HCMC. Cao Lanh Township is the provincial capital; in 2009 the population was 161,292, population density was 1507 per km^2^, and 43.5% of the population was considered to be living in rural areas. The population of Cao Lanh District in 2009 was 200,689, population density was 450 per km^2^ and 93.1% of the population lived in rural areas. This catchment area was selected due to a long-standing research collaboration with the provincial hospital. Four hospitals in HCMC (HTD, Hung Vuong obstetric hospital, District 8 hospital, Children’s Hospital No. 1) and one hospital in Dong Thap province (Dong Thap provincial hospital) participated in the study, and the study protocol was approved by the institutional review boards of these hospitals as well as the Oxford Tropical Research Ethics Committee (OxTREC). The cohort study design is summarized in Figure 2.

### Participant enrolment and collection of baseline data and samples

Women delivering at Hung Vuong obstetric hospital in HCMC or Dong Thap provincial hospital, and resident in the catchment area, were invited to enrol in the cohort and written informed consent was obtained. Women were enrolled either during an antenatal visit in the 9th month of pregnancy or at the time of hospital admission for delivery. Exclusion criteria were girls aged <15 years, those intending to relocate within 12 months and, in HCMC only, women known to be HIV positive. Infants were enrolled within 72 hours of birth, prior to hospital discharge. Enrolment in the cohort began on 1 July 2009 in HCMC and 1 August 2009 in Dong Thap, and is continuing.

Socio-demographic information, obstetric history, infant’s sex, gestational age, delivery method, birth weight and length, breast feeding status, and other post delivery conditions were recorded at baseline. Cord blood (5-20mls) was collected in the labour ward and a maternal blood sample (2mls) was collected within 72 hrs of delivery.

### Routine follow-up

Infants attended scheduled follow-up visits at 2, 4, 6, 9 and 12 months of age, timed to coincide with the delivery of standard Expanded Programme on Immunization (EPI) and private-sector vaccines. In HCMC these vaccines were made available to study participants at the study clinic; in Dong Thap infants received vaccinations independently at commune health posts. In HCMC infants had two additional follow-up visits at 1 and 3 months of age, co-inciding with a routine ‘well-baby’ check-up and EPI vaccinations respectively.

At each visit, information on the infant’s health, weight, height, breastfeeding status, episodes of illness since the last routine visit, and vaccination record was collected in a standardised electronic questionnaire. Infant blood samples (1 ml) were collected by venous blood sampling at 4, 9 and 12 months of age.

Appointments for routine follow-up visits were scheduled in advance, and infants failing to attend a scheduled visit were contacted that day by a study nurse in order to reschedule. If parents were unable to bring infants to the scheduled visit, questionnaires were completed by telephone.

### Loss to follow-up

In the case that a family withdrew from the cohort before the infant’s first birthday, the date and reason for withdrawal were recorded in a standard Outcome CRF in the web database. This CRF was also used to record exit from the cohort due to death, including the date and cause of death.

### Surveillance of infectious disease episodes

Clinical surveillance in the infant cohort aimed to capture all illness episodes within the following syndromes: dengue, respiratory infections, diarrhoeal infections, and hand, foot and mouth disease. In order to capture these syndromes, families were asked to alert a study nurse by telephone whenever an infant was sick and to attend the study clinic at one of the research sites (HTD, District 8 hospital and Dong Thap hospital). On presentation to a study clinic, the treating physician recorded details of illness onset, symptoms, and the presumptive diagnosis into a standard case report form (CRF). Illness episodes which did not fall under any of the above clinical syndromes were not documented. A study nurse collected research specimens as indicated by symptoms and the presumptive diagnosis, as shown in Figure 2. If specimens were taken for routine clinical reasons, then findings from these investigations were recorded in the CRF. Infants returning for follow-up care for a single illness episode had repeat CRFs completed, but research specimens were only collected at the first presentation. A new illness episode was defined as 7 days or more between symptom onset dates.

When an infant was admitted to hospital with one of the defined clinical syndromes, detailed clinical data was collected each day for the duration of hospital stay in a standard CRF. Research specimens were collected on admission only if not already collected at an outpatient consultation. Infants received standard medical care according to hospital guidelines. The only exception to this was the referral for admission of all infants diagnosed with dengue by rapid diagnostic test, in order to permit daily follow-up.

In the case that families could not bring an infant to a study clinic for medical care, we asked that they inform a study nurse by telephone and a CRF was completed over the phone. If the study nurse learned during routine follow-up that an infant had been hospitalised without the prior knowledge of study staff, a study doctor subsequently attempted to abstract detailed clinical data from the hospital notes in order to complete the CRF.

### Laboratory investigations

Routine and clinical samples were stored immediately at 4°C, then transported to the research laboratory once per day, processed and stored at −20°C within 12 hours of collection. A dengue IgM/IgG/NS1 rapid diagnostic test (Dengue Duo; Standard Diagnostics Korea) was performed at the point-of-care for all infants presenting with fever, and later confirmed according to a diagnostic algorithm that includes serotype-specific RT-PCR, NS1 antigen detection by ELISA, and serology on paired plasma specimens by IgM/IgG capture ELISA, as published previously [13,14]. Molecular and serological diagnostic testing for other viral pathogens, including influenza virus, rotavirus, norovirus, and enterovirus, will be performed on batched samples.

### Data management

Data collected at baseline and at routine follow-up visits were recorded in a standardised electronic questionnaire, which stored data directly in an encrypted web-based database. Clinical data recorded at illness presentation in a standard CRF were later double entered into the web database.

Access to the web database is restricted by individual user logins, such that study staff have access only to the forms and datasets that they need and are restricted to read-only or write-only access where appropriate. Electronic forms autofill with patient identifiers, date and time to ensure accuracy, and have built-in data validation tools such as skip patterns, value ranges and dropdown lists to minimise missing and incorrect data. Study participants are identified by a numeric study code that is used on all CRFs and research specimens, and are provided with a study card labelled with this code such that they can be identified when presenting at study clinics.

## Discussion

### Characteristics of the cohort population

**Figure 3:**
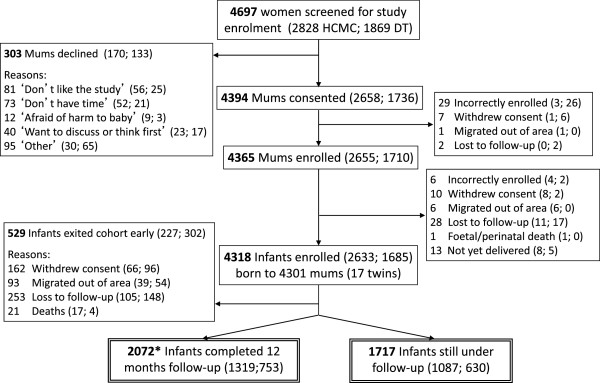
**Flowchart of cohort participants screened, enrolled and followed up between 1 July 2009 (the commencement of enrolment) and 31 August 2011.** Brackets show the numbers by study site (Ho Chi Minh City; Dong Thap).

Between 1 July 2009 and 31 August 2011, 4318 infants born to 4301 women were enrolled in the birth cohort study. The greater number of infants than women represents 17 sets of twins. Figure 3 shows the number of women and infants screened, enrolled, and followed up in the cohort during this period. The proportion of eligible women who consented to partipate was high (93.5%), as was the proportion of enrolled infants who completed the full 12 months of follow-up (84.2%).

Study participants were enrolled from all administrative wards (n = 16 in HCMC and 33 in DT) within the catchment areas. The spatial distribution of participants’ residences is shown in Figure 1B.

Table 1 presents the baseline characteristics of cohort participants enrolled during the first two years of the study. The slight excess of boys over girls reflects the overall sex ratio at birth in Vietnam [15]. Sixty percent of the cohort were first-born children. More than a third of infants in the HCMC cohort were born by caesarean section, compared with only 7% in the DT cohort. This reflects differences in recruitment procedures between the labour wards and the surgical wards at the two hospitals rather than the actual rate of surgical delivery, which averages 30 – 50% at the HCMC hospital and 25% at DT hospital (personal communication). Only a very small proportion of cohort infants were born earlier than 37 weeks gestation (2.7%) or with low birth-weight (<2500 grams; 4.9%), or required admission to the neonatology department prior to hospital discharge (2.4%). Complementary or exclusive feeding with milk formula was common, especially for infants in the HCMC cohort.

**Table 1:** Baseline characteristics of cohort participants

**Characteristic**	**HCMC**	**Dong Thap**	**Total**
**INFANT CHARACTERISTICS (N)**	**2633**	**1685**	**4318**
Sex^2^			
Male	1397 (53.1%)	843 (50.1%)	2240 (51.9%)
Female	1236 (46.9%)	840 (49.9%)	2076 (48.1%)
Gestational age at birth^2^			
<37 weeks	85 (3.2%)	33 (2.0%)	118 (2.7%)
≥ 37 weeks	2548 (96.8%)	1645 (98.0%)	4193 (97.3%)
Method of delivery^2^			
Vaginal	1651 (62.7%)	1561 (92.8%)	3212 (74.4%)
Caesarean	982 (37.3%)	122 (7.2%)	1104 (25.6%)
Birthweight^2^			
<2500 grams	134 (5.1%)	78 (4.6%)	212 (4.9%)
≥ 2500 grams	2499 (94.9%)	1605 (95.4%)	4104 (95.1%)
Siblings			
0	1497 (56.9%)	1081 (64.2%)	2578 (59.7%)
1	917 (34.8%)	498 (29.6%)	1415 (32.8%)
≥ 2	219 (8.3%)	106 (6.3%)	325 (7.5%)
Breastfeeding after birth^3^			
Exclusive breastfeeding	296 (11.2%)	1179 (70.1%)	1475 (34.2%)
Breastfeeding and formula	1895 (72.0%)	491 (29.2%)	2386 (55.3%)
No breastfeeding	442 (16.8%)	12 (0.7%)	454 (10.5%)
Admitted to neonatal ward after birth^2^			
No	2547 (96.7%)	1667 (99.0%)	4214 (97.6%)
Yes	86 (3.3%)	16 (1.0%)	102 (2.4%)
**Mother’s characteristics**			
Ethnicity^2^			
Kinh	2456 (93.3%)	1681 (99.8%)	4137 (95.9%)
Other	176 (6.7%)	3 (0.2%)	179 (4.1%)
Marital status^1^			
Currently married	2578 (97.9%)	1674 (99.3%)	4252 (98.5%)
Other	54 (2.1%)	11 (0.7%)	65 (1.5%)
Highest education level attained^1^			
No school/some primary school	328 (12.5%)	310 (18.4%)	638 (14.8%)
Completed primary school	1362 (51.7%)	987 (58.6%)	2349 (54.4%)
Completed secondary school	545 (20.7%)	233 (13.8%)	778 (18.0%)
Completed university/postgraduate	397 (15.1%)	155 (9.2%)	552 (12.8%)
Occupation^1^			
Stay at home	967 (36.7%)	495 (29.4%)	1462 (33.9%)
Professionals and technicians	182 (6.9%)	141 (8.4%)	323 (7.5%)
Service and sales	897 34.1%)	154 (9.1%)	1051 (24.3%)
Agriculture, craft and trades	490 (18.6%)	489 (29.0%)	979 (22.7%)
Elementary occupations	58 (2.2%)	241 (14.3%)	299 (6.9%)
Other occupations	35 (1.3%)	162 (9.6%)	197 (4.6%)
Unemployed	3 (0.1%)	3 (0.2%)	6 (0.1%)
**Characteristic, cont.**	**HCMC**	**Dong Thap**	**Total**
Number of pregnancies^1^			
1	1129 (42.9%)	938 (55.7%)	2067 (47.9%)
2	836 (31.8%)	476 (28.2%)	1312 (30.4%)
≥ 3	667 (25.3%)	271 (16.1%)	938 (21.7%)
Age at this delivery			
median (IQR)	27 (24 – 32)	25 (21 – 29)	26 (23 – 31)
Age at first childbirth			
median (IQR)	25 (21 – 28)	23 (20 – 25)	24 (21 – 27)
Exposure to passive cigarette smoke^2^			
No	1463 (55.6%)	851 (50.5%)	2314 (53.6%)
Yes	1169 (44.4%)	827 (49.1%)	1996 (46.2%)
Don’t know	0	6 (0.4%)	6 (0.1%)
**Household characteristics**			
Household crowding (people/room)			
median (IQR)	2.3 (1.7 – 3.0)	3.0 (2.0 – 4.0)	2.5 (1.7 – 3.0)
Main source of drinking water^3^			
Bottled	749 (28.5%)	116 (6.9%)	865 (20.0%)
Piped (own residence)	1789 (68.0%)	525 (31.2%)	2314 (53.6%)
Piped (public tap)	45 (1.7%)	173 (10.3%)	218 (5.1%)
Well	46 (1.7%)	21 (1.2%)	67 (1.6%)
Rain/spring/river/stream	3 (0.1%)	848 (50.4%)	851 (19.7%)
Water treated before drinking^2*^			
No	39 (2.1%)	4 (0.3%)	43 (1.3%)
Boiled	1635 (86.8%)	1158 (73.9%)	2793 (80.9%)
Filtered	206 (10.9%)	394 (25.1%)	600 (17.4%)
Other treatment	3 (0.2%)	12 (0.8%)	15 (0.4%)
Main household fuel^4^			
Gas/electricity	2512 (95.5%)	809 (48.1%)	3321 (77.0%)
Wood/coal/straw	46 (1.7%)	869 (51.6%)	915 (21.2%)
Kerosene	73 (2.8%)	5 0.3%)	78 (1.8%)
Keep pigs in household^1^			
No	2619 (99.5%)	1509 (89.6%)	4128 (95.6%)
Yes	13 (0.5%)	176 (10.4%)	189 (4.4%)
Keep birds or poultry in household^2^			
No	2571 (97.7%)	1147 (68.1%)	3718 (86.1%)

^1^ Data missing for 1 participant.

^2^ Data missing for 2 participants.

^3^ Data missing for 3 participants.

^4^ Data missing for 4 participants.

* Of those who do not use bottled water.

### Study strengths

To our knowledge, this is the first longitudinal prospective study of infectious diseases in the first year of life in southeast Asia. A major strength of the study is the ability to link baseline sociodemographic and geolocation data on the mothers and their infants with clinical and laboratory data from acute illness episodes during the first year of life, and also with longitudinal serological data, beginning with cord blood collected at birth. This design permits investigations of individual, environmental, virological, and immunological determinants of infection and disease in infants. Specifically, we are able to investigate the duration and nature of passive protection against infectious diseases by maternal antibody acquired at birth, and to characterise the adaptive immune response in infants following primary infection. This has application for understanding force of infection, correlates of protection, and the potential role for maternal vaccination in protecting infants against some infectious diseases.

A primary research question that informed the design of this cohort study is to identify correlates of immunity and pathogenesis in infants and children exposed to dengue virus infections. Pre-existing non-neutralising antibodies are thought to drive the observed increased incidence of severe disease following secondary, heterotypic dengue virus infections, through a mechanism of antibody-dependent enhancement [16]. Indirect evidence suggests that the same mechanism may explain the age-related epidemiology of dengue in infants in endemic countries, where symptomatic dengue cases occur most frequently between the ages of 4 to 10 months, coinciding with the time at which maternally derived neutralising antibodies to DENV become undetectable but non-neutralising viron-binding antibodies remain detectable [6,7]. However the degree to which in vitro neutralizing antibody activity, or enhancing antibody activity, correlates with dengue immunity or disease is still uncertain [17-19]. This was strikingly apparent in the recently published results of the first efficacy trial of a dengue vaccine candidate, in which there was no clinical efficacy against the predominant DENV-2 serotype, despite satisfactory immunogenicity as defined by serotype-specific neutralising antibody seropositivity and geometric mean titres [20]. Our study will provide the opportunity to assess directly the way in which pre-existing multitypic anti-DENV antibody shapes infection outcome, as well as to characterise longitudinally the adaptive immune response following a primary DENV infection.

### Study limitations

Our cohort was not designed as a population-representative sample; for pragmatic reasons, we restricted our catchment areas to one urban and one mixed urban/rural district where we had existing research collaborations, and where the burden of dengue and other infectious diseases is known to be high. This may limit the generalisability of the epidemiological findings, but is unlikely to affect the external validity of the immunological and virological analyses which are the focus of our primary research questions. The inclusion of both urban and semi-rural cohorts is a strength of our study, since the disease burden and relevant exposures might differ substantially between these settings. Enrolment at obstetric hospitals rather than in the community might skew our sample towards women of higher socio-economic status, although in Vietnam the vast majority of births occur in medical facilities so this is unlikely to be a major source of bias.

### Internal validity

The two major challenges for our study are minimising loss to follow-up and maximising ascertainment of acute illness episodes. To date our cohort retention has been high, with 84% of infants completing the 12-month follow-up period. We acknowledge that we will not be able to capture every acute illness episode in our surveillance, and therefore that estimates of disease incidence will be underestimates. The problem of under-ascertainment of acute illness episodes does have the potential to introduce bias into any analyses that compare clinical endpoints between subgroups, where those subgroups are likely to differ systematically in their completeness of case ascertainment – for example, between study sites or socioeconomic groups or infants with different baseline health status. This limitation will be considered when interpreting any subgroup-specific estimates of disease incidence.

### Future plans

Enrolment in the infant cohort is ongoing, and from 1 August 2012 enrolment also began in an extension phase of the cohort. This phase will retain approximately 1000 infants in the cohort from their first birthday until six years of age, but with a reduced schedule of routine data and blood collection every six months and no acute illness surveillance or sampling. Our current research interests are focused on common viral infections of childhood in Vietnam, namely dengue, influenza, and hand foot and mouth disease, and viral and bacterial enteric diseases. However the study provides a platform that could easily accommodate additional endpoints in the future, including non-communicable diseases such as asthma, allergy and obesity, which are likely to become increasing health problems in this setting.

## Competing interests

The authors declare that they have no competing interests.

## Authors’ contributions

KLA participated in the design of the study, oversaw the study co-ordination, data collection and analysis, and wrote the manuscript. NMN participated in the design of the study and co-ordinated the collection of clinical data in HCMC. NTVT contributed to study co-ordination and data monitoring and cleaning. NTH oversaw participant enrolment and routine follow-up. HLN contributed to data cleaning and analysis and critically reviewed the manuscript. NTHT, PTTH and LBL oversaw the collection of clinical data at collaborating hospitals. NVVC contributed to the design and co-ordination of the study. CPS conceived of and designed the study, and critically reviewed the manuscript. All authors read and approved the final manuscript.

## Authors’ information

Additional members of the Birth Cohort Study team who contributed to the design and implementation of the study are as follows: Nguyen Thanh Tien, Nguyen Thi Hong Van, Bridget Wills, Jeremy Farrar (Oxford University Clinical Research Unit); Nguyen Van Truong (Hung Vuong Hospital); Mai Ngoc Lanh, Pham Thi Hong Phuong, Vo Thi Bich Ngoc, Dang Thi Ngoc Ha, Le Thi Phuong (Dong Thap Hospital); Nguyen Thi Thanh Thuy (District 8 Hospital); Nguyen Thanh Hung, Nguyen Minh Tuan (Children’s Hospital No. 1 HCMC).

## Pre-publication history

The pre-publication history for this paper can be accessed here:

http://www.biomedcentral.com/1471-2458/13/937/prepub
